# Chemotherapy plus bevacizumab versus chemotherapy plus cetuximab as first-line treatment for patients with metastatic colorectal cancer: Results of a registry-based cohort analysis

**DOI:** 10.1097/MD.0000000000004531

**Published:** 2016-12-23

**Authors:** Long Bai, Feng Wang, Zhe-zhen Li, Chao Ren, Dong-sheng Zhang, Qi Zhao, Yun-xin Lu, De-shen Wang, Huai-qiang Ju, Miao-zhen Qiu, Zhi-qiang Wang, Feng-hua Wang, Rui-hua Xu

**Affiliations:** a Department of Medical Oncology, Sun Yat-sen University Cancer Center; b Sun Yat-sen University Cancer Center; State Key Laboratory of Oncology in South China; Collaborative Innovation Center for Cancer Medicine, Guangzhou; c State Key Laboratory of Biocontrol, School of Life Sciences, Sun Yat-sen University, Guangzhou, Guangdong, P. R. China.

**Keywords:** bevacizumab, cetuximab, Chinese population, first-line treatment, metastatic colorectal cancer, peritoneal metastasis

## Abstract

The present observational cohort study was designed to elucidate the efficacy and safety profile of bevacizumab or cetuximab with chemotherapy as the first-line treatment in Chinese patients with metastatic colorectal cancer (mCRC). Clinical data were collected from a single-center registry study where mCRC patients received first-line fluoropyrimidine-based chemotherapy combined with either bevacizumab (188 patients with *KRAS* wild-type or mutated tumors) or cetuximab (101 patients with *KRAS* wild-type tumors) between January 2009 and December 2013. The Kaplan–Meier method was used for survival analysis. Cox proportional hazards model was used for estimating the prognostic and predictive values of clinicopathological characteristics. No statistically significant difference was observed between the bevacizumab and cetuximab groups in terms of median progression-free survival (PFS) (10.6 vs 8.7 months, *P* = 0.317), median overall survival (OS) (27.7 vs 28.3 months, *P* = 0.525), or overall response rate (43.1% vs 53.5%, *P* = 0.108). For the subset of patients with peritoneal dissemination, bevacizumab-based triplet appears to be superior to cetuximab-based triplet as measured by PFS (9.6 vs 6.1 months) and OS (26.3 vs 12.7 months), but not for patients without peritoneal dissemination (PFS, 10.6 vs 9.1 months; OS, 27.9 vs 30.7 months) (all unadjusted and adjusted interaction *P* < 0.05). Our study suggests that bevacizumab- or cetuximab-based regimens have similar effectiveness as first-line treatment of mCRC in Chinese population. Patients with peritoneal dissemination were likely to gain more benefit from bevacizumab than cetuximab treatment. Future prospective studies are required to further confirm these results.

## Abstract 2

Supplemental Digital Content is available in the text

## 1 Introduction

Colorectal cancer is the third most commonly diagnosed cancer in males and the second in females worldwide. Approximately 15% of colorectal cancer (CRC) patients would present with metastatic disease at diagnosis, and a further 40% to 50% would continuously develop metastases through the course of disease.^[1,2]^ The introduction of bevacizumab (Avastin; Hoffmann-Laroche, Basel, Switzerland), a humanized monoclonal antibody that inhibits tumor angiogenesis by neutralizing vascular endothelial growth factor (VEGF), together with cetuximab (C225; Merck KGaA, Darmstadt, Germany), a chimeric antiepidermal growth factor receptor (EGFR) antibody that blocks several cell signaling pathways activation, has deeply modified the handling of metastatic colorectal cancer (mCRC).^[3–5]^ Currently, fluoropyrimidine-based chemotherapy in combination with either bevacizumab or cetuximab has been widely adopted as the standard of care as first-line treatment for mCRC patients.^[6–10]^ While mutations in the *RAS* oncogene well predicts resistance to anti-EGFR agents,^[11–14]^ however, no biomarker can predict the magnitude of benefit from bevacizumab or cetuximab in the *RAS* wild-type population so far.^[15–17]^ Their optimal use in terms of patient selection, drug combinations, and regimen sequences remains inconclusive.^[18–21]^

Two phase III clinical trials have compared bevacizumab with cetuximab in first-line mCRC treatment in a head-to-head setting. GERMAN AIO KRK-0306 (FIRE-3) study compared FOLFIRI with bevacizumab or cetuximab in 592 *KRAS* wild-type patients. A significantly prolonged overall survival (OS) was observed (28.7 vs 25.0 months; hazard ratio [HR] = 0.77, *P* = 0.017) besides similar progression-free survival (PFS) (10.0 vs 10.3 months; HR = 1.06, *P* = 0.547) and overall response rate (ORR) (62% vs 58%, *P* = 0.183) in cetuximab group versus bevacizumab group.^[22]^ Recent analyses demonstrated even more pronounced OS benefit in all *RAS* wild-type patients (33.1 vs 25.9 months, *P* = 0.010), which favored the cetuximab combination.^[23]^ By contrast, in a larger trial, CALGB80405 (n = 1137), bevacizumab or cetuximab combined with chemotherapy conferred comparable outcomes in PFS (10.8 vs 10.4 months; HR = 1.04, *P* = 0.55) and OS (29.0 vs 29.9 months; HR = 0.92, *P* = 0.34) in *KRAS* wild-type population. Recent updated PFS (11.4 vs 11.3 months) and OS (32.0 vs 31.2 months) results in *RAS* wild-type patients also showed no significant difference between the 2 arms.^[24]^

The presence of a benefit in OS but lack thereof in PFS and ORR for the cetuximab arm in FIRE-3 trial, and the discrepancy of OS between these 2 trials caused confusion among oncologists.^[25,26]^ Moreover, the efficacy and safety profile of bevacizumab and cetuximab in Chinese mCRC patients has not been assessed in previous randomized controlled trials. Hence, this single-center registry study was designed to compare bevacizumab (in patients with either *KRAS* wild-type or mutated tumors) with cetuximab (in patients with *KRAS* wild-type tumors) in the first-line treatment for Chinese mCRC patients.

## 2 Patients and methods

### 2.1 Patients and treatment

The study cohort was developed from a single-center registry, which evaluated the efficacy and safety profile of bevacizumab or cetuximab combined with first-line chemotherapy in Chinese mCRC patients treated at Sun Yet-sen University Cancer Center from 2009 January to 2013 December. Histologically proven stage IV (locally advanced or metastatic) CRC patients, who have consecutively received at least 2 courses of bevacizumab-based (patients with either *KRAS* wild-type or mutated tumors) or cetuximab-based (patients with KRAS wild-type) triplet biochemotherapy as their first-line treatments were enrolled. Informed consent was obtained from all individual participants included in the study. Information collected from the registry data source included baseline demographic and disease characteristics, laboratory data, dates and doses of chemotherapy and target therapy, imaging scan results, adverse drug events, and data on survival.

Enrolled patients were administered mFOLFOX-6,^[27]^ XELOX,^[28]^ or FOLFIRI^[29]^ in combination with cetuximab 400 mg/m^2^ taken at the first dose and followed by 500 mg/m^2^ on biweekly schedules or 750 mg/m^2^ on triweekly schedules, or in combination with bevacizumab 5 mg/kg on biweekly schedules or 7.5 mg/kg on triweekly schedules. The backbone chemotherapy regimens, duration of biochemotherapy, and the introduction of maintenance therapy (monotherapy of capecitabine, bevacizumab or cetuximab, or bevacizumab combined with capecitabine) were at the physician's discretion. A new drug adding to a regimen within 28 days of the start of a regimen was considered an addition to the existing line, rather than a change in line of therapy. Similarly, withdrawal of a single drug from a combination regimen was not considered as a new line of therapy.

The registry has been carried out in compliance with the Helsinki declaration and has been approved by the Institutional Review Board (IRB) and Human Ethics Committee. The study protocol for the collection of individual patient information was approved by the IRB.

### 2.2 Assessment

PFS was measured from the initiation of biochemotherapy to disease progression or death from any cause. OS was defined as the time from the start of biochemotherapy to death from any cause. For patients who were alive at final analysis, data on survival were censored at the last contact. Tumor response was assessed by the investigators according to the Response Evaluation Criteria in Solid Tumors version 1.1. ORR was defined as complete response (CR) plus partial response (PR) as best response. Disease control rate (DCR) was defined as CR, PR plus stable disease as best response. The US National Cancer Institute's Common Terminology Criteria for Adverse Events, version 3.0, was used to grade each toxicity event. The probable or possible cause of clinically relevant event of bevacizumab was also recorded. Serious adverse events (SAEs) were those leading to prolonged hospitalization, life-threatening events, or death.

### 2.3 Statistical analysis

The association of safety data, response rates, or patient characteristics between 2 arms was examined by the Chi-square test, Fisher exact probability test, or the Mann–Whitney test if required. The Kaplan–Meier method and log-rank test were performed to estimate PFS and OS and examine the survival differences between treatment groups, respectively.

In order to estimate the prognostic value of baseline clinicopathological features, Cox proportional hazards models were used in univariate and multivariate analyses and to generate the HR and corresponding 95% confidence intervals (CIs). Subsequently, in order to define a subgroup of patients who probably obtain different benefit from bevacizumab- and cetuximab-based regimens, interaction Cox-Wald test was performed according to clinical stratifying factors and treatment arms. Additional multivariable Cox regression model adjusted for potential prognostic variables were also run. A backward elimination algorithm was used to select significant covariates in multivariate analyses. The significance level for selecting these covariates was set at 0.157, which is about equivalent to Akaike information criteria.^[30]^ SPSS 19.0 statistical software package (SPSS Inc., Chicago, IL) and STATA 12.0 (Stata, College Station, TX) were used in statistical analyses.

## 3 Results

### 3.1 Enrollment and follow-up

**Figure 1:**
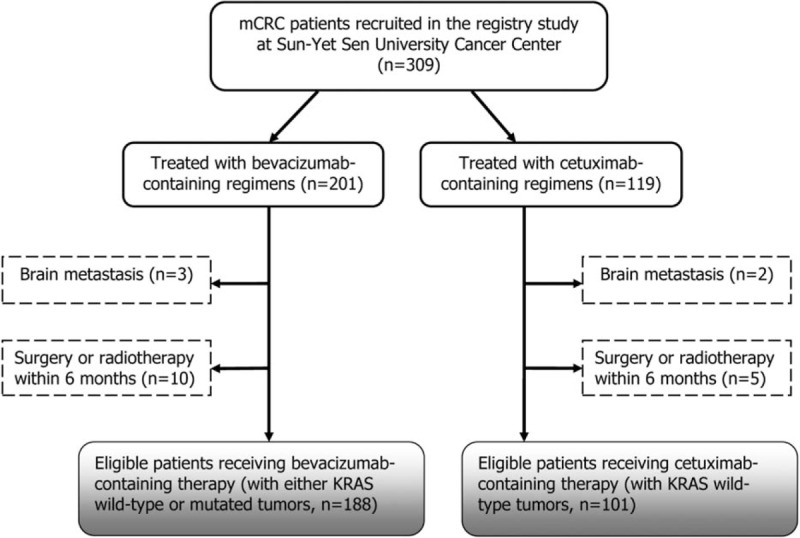
Study patient disposition.

From the registry data source, a total of 309 mCRC patients who initiated bevacizumab or cetuximab as part of their first-line treatment from January 2009 to December 2013 were screened for study eligibility. Further screening of subjects based on verification of sufficient evaluable data determined 289 patients for final analysis. Among these patients, 188 (65.1%) patients received bevacizumab-based triplet and 101 (34.9%) received cetuximab-based triplet (Fig. 1). Until the last follow update, October 1, 2015, the median follow-up time was 21.3 months (range, 0.3–48.2 months). In the bevacizumab group and cetuximab group, 152 (80.9%) and 88 (87.1%) patients had documented progressive disease (PD), 93 (60.6%) and 57 (56.4%) patients had died, 21 (11.2%) and 10 (9.9%) patients were progression free and alive, 9 (4.8%) and 9 (8.9%) patients were lost to follow-up, respectively.

### 3.2 Patient characteristics

Main baseline demographic and clinical characteristics were generally balanced across the 2 groups, only with a few exceptions (Table 1). First, in the bevacizumab group, 50 (26.6%) patients had *KRAS*-mutated tumors, 63 (33.5) patients had *KRAS* wild-type, and 75 (39.9%) patients did not have their *KRAS* status tested. Cetuximab was only administered in patients with *KRAS* wild-type tumors. Second, the percentage of patients receiving curative-intent metastasectomy (27.7% vs 13.9%, *P* = 0.007) was higher in the cetuximab group than in the bevacizumab group. Lastly, patients in the bevacizumab group had a higher proportion of peritoneal metastasis (30.5% vs 18.8%, *P* = 0.036) and intact primary tumor (30.5% vs 18.8%, *P* = 0.007) than those in the cetuximab group at baseline evaluation.

**Table 1:**
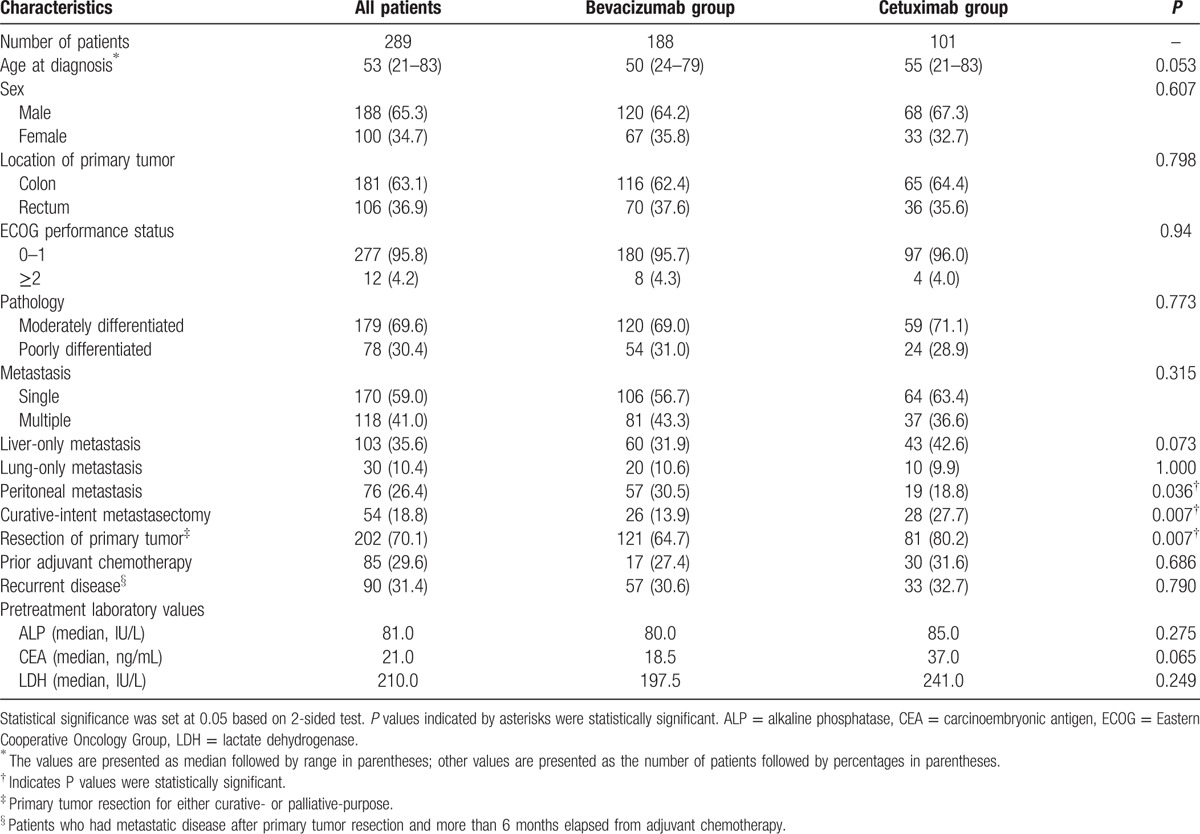
Baseline clinical characteristics across treatment groups.

### 3.3 Treatment patterns

Median total duration of chemotherapy, exposure to second-line therapy or third-line therapy, and percentage of all 3 active chemotherapy agents for metastatic disease (oxaliplatin, irinotecan, and 5-FU/capecitabine) were equivalent between the 2 arms (Table 2). However, we still observed some discrepancies between the 2 arms in treatment patterns. First of all, the selection of combined chemotherapy agents differed between the 2 groups. More patients in the bevacizumab group were treated with oxaliplatin-based chemotherapy than irinotecan-based chemotherapy (60.2% vs 39.8%), while a reverse trend was observed in the cetuximab group (37.1% vs 62.9%) (Chi-square test, *P* < 0.001). Next, the median first-line treatment duration was longer in the bevacizumab group than in the cetuximab group (antibodies, 4.5 vs 3.0 months, *P* = 0.005; backbone chemotherapies, 5.5 vs 4.0 months, *P* = 0.004). Besides, more patients in the cetuximab group halted the antibody before first PD than those in the bevacizumab group (66.3% vs 53.2%, *P* = 0.043). Similarly, more patients continued antibody administration beyond first PD in the bevacizumab group than in the cetuximab group (24.6% vs 8.4%, *P* = 0.001). In contrast, significantly more patients in cetuximab group were exposed to crossover antibody than in bevacizumab group both in second-line (31.6% vs 11.7%, *P* < 0.001) and during the course of disease (40.4% vs 18.2%, *P* < 0.001). None of the patients in both groups received panitumumab in any line of treatment. In addition, more patients received maintenance therapy in the bevacizumab group than in the cetuximab group (34.2% vs 12.0%, *P* < 0.001).

**Table 2:**
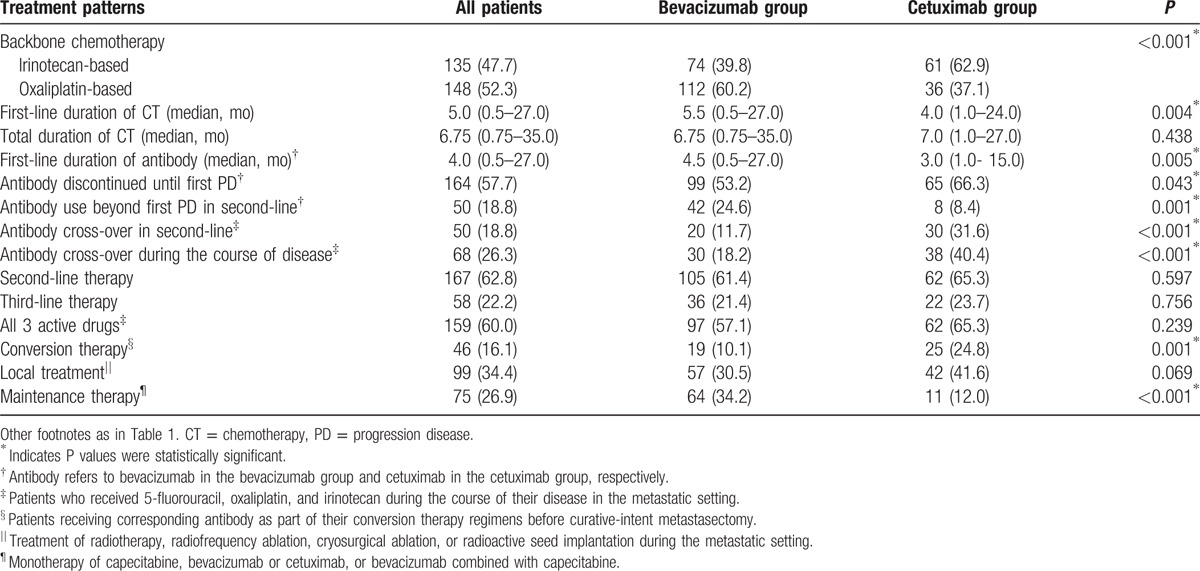
Treatment patterns across treatment groups.

### 3.4 Effectiveness outcomes

**Figure 2:**
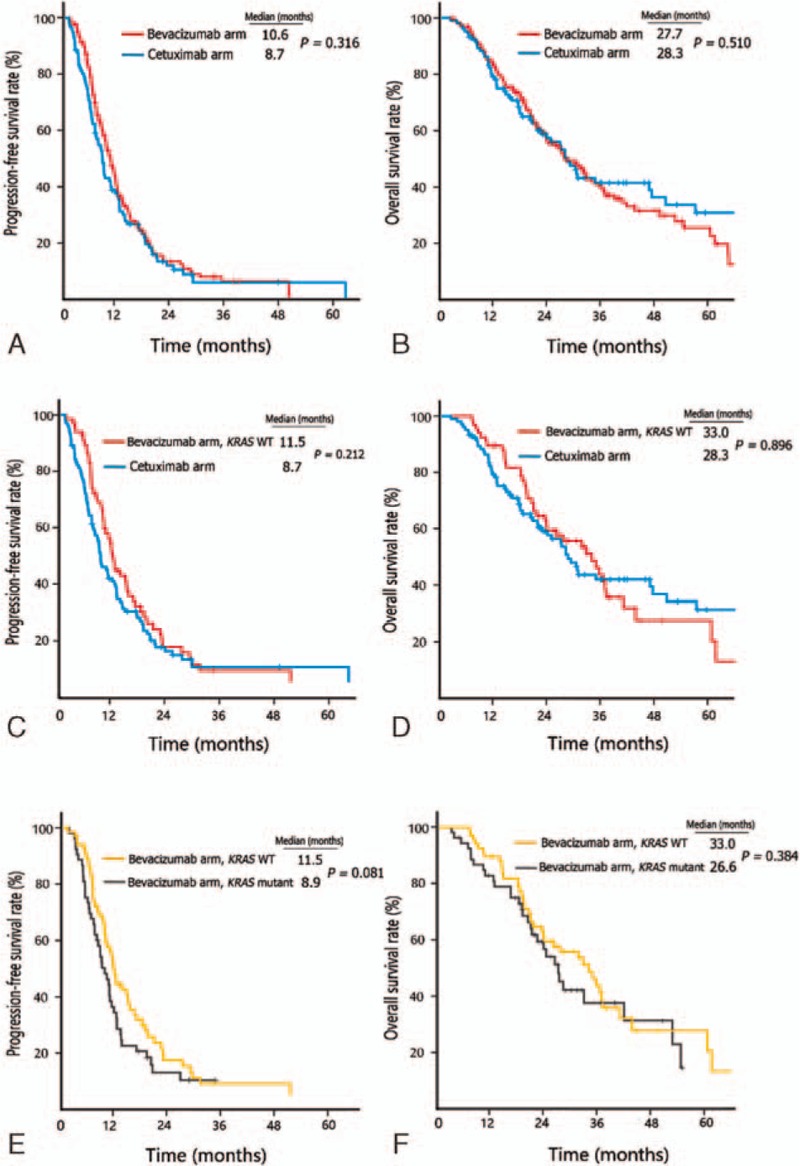
Kaplan–Meier survival estimates of patients across different subgroups. (A) Progression-free survival (PFS) curves of all patients. (B) Overall survival (OS) curves of all patients. (C) PFS curves of patients with *KRAS* wild-type tumors. (D) OS curves of patients with KRAS wild-type tumors. (E) PFS curves of patients in the bevacizumab group. (F) OS curves of patients in the bevacizumab group. *KRAS* WT = *KRAS* wild-type.

Median PFS was 10.6 months (95% CI 9.3–11.9 months) in the bevacizumab group and 8.7 months (95% CI 7.5–9.9 months) in the cetuximab group (HR 1.15, 95% CI 0.88–1.50, *P* = 0.316) (Fig. 2A). Median OS was 27.7 months (95% CI 22.7–32.7 months) in the bevacizumab group versus 28.3 months (95% CI 22.7–33.9 months) in the cetuximab group (HR 0.90, 95% CI 0.65–1.24, *P* = 0.510) (Fig. 2B). For patients with wild-type *KRAS*, no significant differences of PFS (11.5 vs 8.7 months, *P* = 0.212) and OS (33.0 vs 28.3 months, *P* = 0.896) were observed in the bevacizumab and cetuximab groups (Fig. 2C and D). Of note, in the bevacizumab group, wild-type *KRAS* versus mutant *KRAS* was associated with a trend toward prolonged PFS (11.5 vs 8.9 months, *P* = 0.081) and OS (33.0 vs 26.6 months, *P* = 0.384) (Fig. 2E and F).

A total of 95.1% (275/289) patients were assessable for tumor response (Table 3). Cetuximab-containing regimens induced a trend of higher ORR compared with bevacizumab-containing regimens (53.5% vs 43.1%, *P* = 0.108). Besides, both treatment groups have achieved a high DCR (87.5% in the bevacizumab group vs 88.1% in the cetuximab group).

**Table 3:**
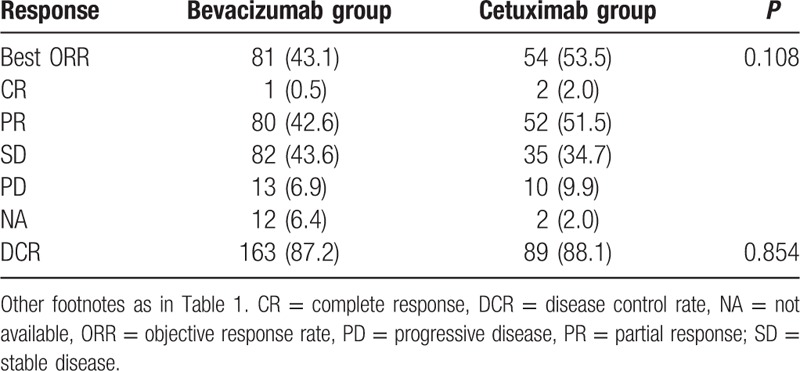
Response rate to first-line treatment across treatment groups.

### 3.5 Curative-intent metastasectomy

After biochemotherapies, curative-intent metastasectomy was carried out in 10.1% (19/188) of patients in the bevacizumab group and 24.8% (25/101) in the cetuximab group. For patients with liver- or lung-confined disease, cetuximab-based regimens were associated with a relatively higher ORR (69.6% vs 59.3%, *P* = 0.559) and a higher conversion rate to resectability (46.3% [25/54] vs 28.8% [19/66], *P* = 0.058) compared with bevacizumab-based regimens. Patients who subsequently underwent curative-intent metastasectomy showed comparable outcomes between the bevacizumab and cetuximab groups in terms of median PFS (12.4 vs 13.9 months, *P* = 0.979) and OS (55.3 vs 71.6 months, *P* = 0.402). Long-term survival was achieved in the subset of patients who received curative-intent metastasectomy compared with those who did not undergo metastasectomy in both the bevacizumab group (55.3 vs 24.2 months, *P* = 0.008) and the cetuximab group (71.6 vs 20.4 months, *P* < 0.001).

### 3.6 Prognostic and predictive value of clinical factors

In the bevacizumab group, performance status ≥2, poorly differentiated tumor, absence of metastasectomy, and elevated baseline serum carcinoembryonic antigen (CEA) or lactate dehydrogenase levels were identified as independent poor prognostic factors for OS by univariate and multivariate analyses. While in the cetuximab group, the absence of metastasectomy and elevated CEA level was independently associated with poorer OS (*P* < 0.05 for all, Supplementary tables S1 and S2).

**Figure 3:**
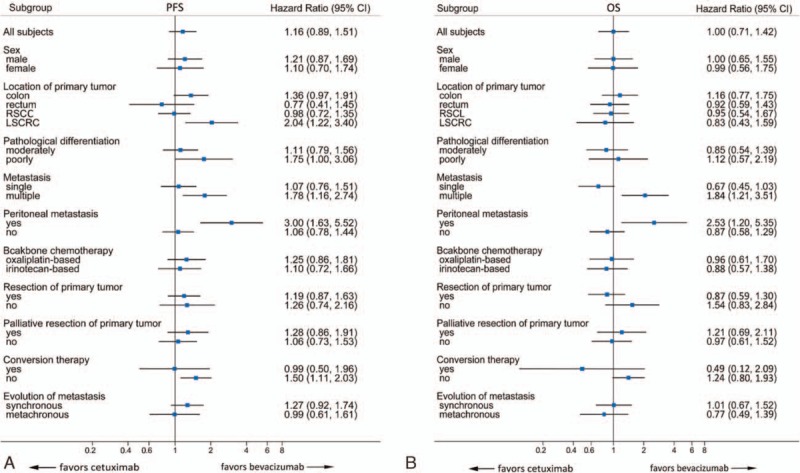
Forest plots of treatment hazard ratios (bevacizumab-based regimens versus cetuximab-based regimens) for (A) progression-free survival and (B) overall survival according to subgroups of baseline demographic and stratification variables. 95% CI = 95% confidence interval, HR = hazard ratio, LSCRC = left-sided colorectal cancer, OS = overall survival, PFS = progression-free survival, RSCC = right-sided colon cancer.

When patients were divided into subgroups according to stratification factors such as age, gender, performance status, site of primary tumor, histological subtypes, number and sites of metastasis, resection of primary tumor, metastasectomy, chemotherapy regimen, presence of synchronous or metachronous metastasis, and basal biochemistry marker level, no particular subgroup of patients could be identified to benefit more from cetuximab versus bevacizumab or vice versa by interaction Cox-Wald test (Fig. 3).

**Figure 4:**
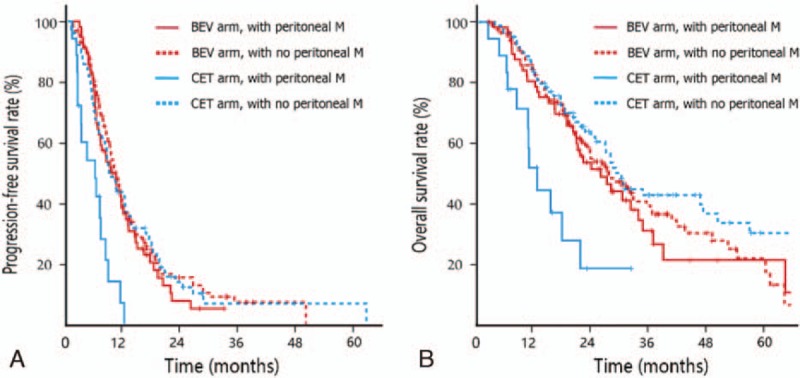
The predictive values of peritoneal metastasis for progression-free survival (A) and overall survival (B) were presented by Kaplan–Meier curves stratified according to presence/absence of peritoneal metastasis and treatment arms. Peritoneal M = peritoneal metastasis.

However, peritoneal involvement was identified as a significant predictor for the benefits from the 2 target agents. For patients with peritoneal metastasis, bevacizumab-based triplet appears to be superior to cetuximab-based triplet as measured by median PFS (9.6 vs 6.1 months; HR 3.00, 95% CI 1.63–5.52, *P* < 0.001) and OS (26.3 vs 12.7 months; HR 2.53, 95% CI 1.20–5.35, *P* = 0.006), while PFS (median PFS, 10.6 vs 9.1 months; HR 1.06, 95% CI 0.78–1.44, *P* = 0.775) and OS (median OS, 27.9 vs 30.7 months, HR 0.87, 95% CI 0.58–1.29, *P* = 0.187) were similar in the bevacizumab group and the cetuximab group. After adjusted for potentially prognostic factors, a significant interaction was observed between treatment effectiveness of the 2 groups and the involvement of peritoneal metastasis, both for PFS (unadjusted interaction *P* = 0.001 and adjusted interaction *P* = 0.024) and OS (unadjusted interaction *P* = 0.005 and adjusted interaction *P* = 0.025) (Table 4 and Fig. 4). Importantly, removing cases where patients received curative-intent metastasectomy did not alter the above results (Supplementary table S3).

**Table 4:**
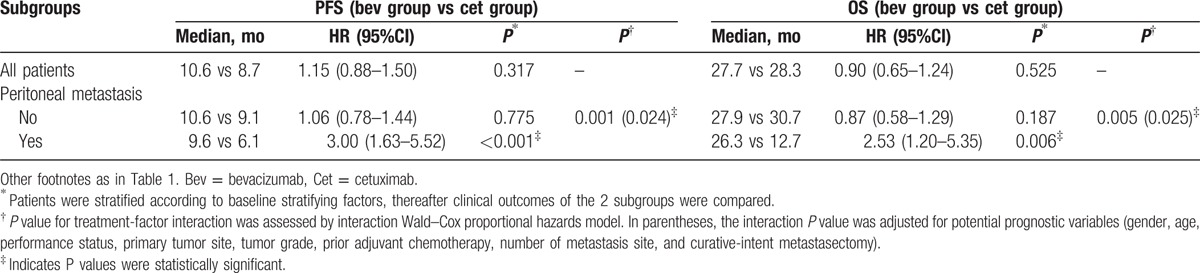
Correlations between baseline stratifying factors and clinical outcomes.

### 3.7 Tolerability

The overall incidence of grades 3 to 4 adverse event (AE) was similar between the 2 groups, which was 41.0% in the bevacizumab group versus 41.6% in the cetuximab group. The most common grades 3 to 4 AEs were neutropenia (24.6%), asthenia (13.1%), and emesis (12.1%), with comparable incidence across the 2 groups, except that acneiform exanthema was more frequently caused by cetuximab administration than bevacizumab (9.9% vs 2.7%, *P* = 0.012) (Supplementary table S4). There were no significant differences between the 2 groups in terms of SAEs (bevacizumab group, 15.4%; cetuximab group, 15.8%). Bevacizumab-related AEs occurred in 13 patients (6.9%), mostly hemorrhage (n = 7, 3.7%). Fatal AEs were rare (n = 1, 0.6%), 1 case in the bevacizumab group presented with a central nervous system bleeding 2 months after the last dose of bevacizumab. The all-cause 60-day mortality was 0% in both groups (Supplementary table S4).

As for bevacizumab-specific AEs, hemorrhage cases were reported in 22 (11.7%) patients in the bevacizumab group. Total 15 bleeding events (8.0%) required temporary or permanent discontinuation of bevacizumab administration. No surgery-related grades 3 to 4 bleeding events were observed in patients who received perioperative bevacizumab treatment (n = 71). One case of postoperative wound dehiscence and 1 case of intestine perforation was reported, both of which required another emergency surgery. Besides, hypertension, proteinuria and arterial/venous thrombosis were most mild and medically manageable (Supplementary table S5).

## 4 Discussion

Due to advances in drug development over the last 2 decades, median OS can now be as long as 30 months in selected mCRC patients.^[31–33]^ However, significant challenges regarding the optimization of targeted therapies still remain.^[34]^ In the present evaluation of Chinese mCRC patients from a real-world registry, first-line bevacizumab- and cetuximab-based triplets were well tolerated, and yielded similar effectiveness in PFS (10.6 vs 8.7 months; HR = 1.15, *P* = 0.317), OS (27.7 vs 28.3 months; HR = 0.90, *P* = 0.525), and ORR (43.1% vs 53.5%, *P* = 0.108).

In previous studies, patients treated with first-line bevacizumab-based regimens have consistently experienced an OS in the range of 23.0 to 25.9 months in community-based patient populations,^[35–37]^ and an OS of 24.5 to 29.0 months in randomized controlled trials (RCTs).^[38–40]^ On the other hand, *KRAS* wild-type mCRC patients receiving cetuximab-based regimens have demonstrated an OS in the range of 22.8 to 29.9 months in RCT settings.^[41–43]^ Published information was limited in first-line cetuximab-based regimens in community-based settings. In the present study, the median OS of 27.7 months achieved in the bevacizumab group and 28.3 months in the cetuximab group were comparable with prior studies, which was encouraging as no exclusions were made on the items of Eastern Cooperative Oncology Group performance status, age, or organ functions—exclusion criteria were regularly used in previous RCTs.

While neither antibody demonstrated a clearly superior outcome in the present analysis, the OS curves split after 2.5 years, about 1.5 years after disease progression on first-line therapy, thereafter appearing to favor the cetuximab combination (Fig. 2B). Similar results were also observed in FIRE-3 and PEAK trials.^[22,44]^ In spite of several discrepancies observed in treatment patterns, the median total chemotherapy duration, exposure to second- or third-line therapy, and percentage of all 3 active chemotherapy agents were equivalent between the 2 arms and therefore unlikely to influence the OS. One possibility is that the OS results were confounded by the uneven distribution of patients who underwent curative-intent metastasectomy between the cetuximab and bevacizumab groups (27.7% vs 13.9%, *P* = 0.007). Additional follow-up may be needed to further clarify the situation.

In our study, patients receiving the cetuximab-based triplet versus bevacizumab-based triplet had a higher conversion rate to resectability (46.3% vs 28.8%, *P* = 0.058) in liver- or lung-confined diseases. In addition, the subset of patients who subsequently underwent curative-intent metastasectomy were rendered a long-term survival (median OS of 71.6 months in the cetuximab group vs 55.3 months in the bevacizumab group, *P* = 0.402). Though most patients with single-metastasis disease were reviewed by experienced medical oncologists or hepatobiliary surgeons at our cancer center, formal resectability criteria were not required in the study protocol, which might fail in identifying initial resectable diseases and confound the patient selection between the 2 groups. Therefore, further randomized trials are needed to define the optimal targeted treatment strategy for conversion therapy.

It was of interest to observe that in the bevacizumab group, wild-type versus mutant-type *KRAS* genotype was associated with a trend toward prolonged PFS (11.5 vs 8.9 months, *P* = 0.081) and OS (33.0 vs 26.6 months, *P* = 0.362) (Fig. 2E and F). Since evidence from randomized trials was rather limited, the prognostic value of *KRAS* mutation in bevacizumab treatment remains controversial.^[45–47]^ Taking into consideration that the treatment patterns (frequencies of second-line chemotherapy, continuous bevacizumab usage before PD, bevacizumab continuation beyond PD, maintenance therapy, and curative-intent metastasectomy) were virtually identical between these 2 subgroups, the different survival may be partially explained by the higher percentage of patients who received subsequent crossover cetuximab in the *KRAS* wild-type subgroup than in the *KRAS* mutant subgroup (49.2% vs 2.2%, *P* < 0.001). Consequently, when *KRAS* mutant patients were excluded from the bevacizumab group, receiving bevacizumab was associated with a comparatively longer PFS (11.5 vs 8.7 months, *P* = 0.212) and OS (33.0 vs 28.3 months, *P* = 0.936) (Fig. 2C and D) compared with cetuximab in *KRAS* wild-type patients (Fig. 2C and D), which was in contrast to the results in FIRE-3 trial.^[22]^ However, it should be noted that *KRAS* wild-type patients were limited in the bevacizumab group (n = 63). It needs to be cautious, as oncologists, to interpret the data. Given these, the prognostic significance of *KRAS* genotype for bevacizumab treatment in mCRC needs further investigation.

The present study demonstrated that, for patients with peritoneal metastases, the results appear to favor bevacizumab combinations over cetuximab combinations (PFS 9.6 vs 6.1 months; OS 26.3 vs 12.7 months). Peritoneal spread occurs in 10% to 15% of mCRC patients.^[48]^ It causes the terminal stage of colorectal cancer; diminishes quality of life by ascites retention, malnutrition, and intestinal obstruction; and is associated with a poor prognosis.^[49,50]^ Nevertheless, effective management against peritoneal metastasis has not yet been established. Some studies suggest that alternative routes of bevacizumab administration may be helpful, with reports of bevacizumab intraperitoneal perfusion leading to significant symptomatic benefit for ovarian cancer patients with peritoneal dissemination.^[51,52]^ However, the therapeutic effect of bevacizumab for peritoneal disease in mCRC has been poorly described. The AGITG MAX trial,^[53]^ the only randomized Phase III trial where the outcomes of mCRC patients with peritoneal metastasis receiving bevacizumab treatment were presented, together with 2 registry-based studies—TRACC^[54]^ and NSWCC,^[55]^ have showed that the addition of bevacizumab to first-line chemotherapy was associated with prolonged PFS within the peritoneal subgroup. But the OS benefits were not consistently seen across studies.

VEGF levels in peritoneal metastasis sites have been reported to be remarkably elevated,^[56,57]^ which might enhance angiogenesis and vascular permeability in the abdominal wall and contributes to the establishment of malignant ascites.^[58,59]^ Thus, we hypothesize that VEGF inhibitors can be more efficacious than EGFR inhibitors against peritoneal tumors that are often VEGF independent and actively vascularizing.^[60,61]^ This hypothesis was supported by an animal model where the neutralization of the biological activity of VEGF by bevacizumab could inhibit the outgrowth of new blood vessel and subsequently suppress the peritoneal dissemination from gastric cancer. These results could explain, at least partially, conflicting results among clinical trials concerning cetuximab and bevacizumab effectiveness in mCRC,^[25,26]^ underscoring the need to report the proportion of patients with peritoneal disease.

It was worth mentioning that the incidence of bevacizumab-related AE, gastrointestinal perforation, was not being impacted by the presence or absence of peritoneal disease following bevacizumab administration (0% [0/57] vs 1.5% [2/131], *P* = 0.99). The result was in accordance with the findings of previous studies both in trial and nontrial settings.^[35,36,46,62,63]^ Thus, we recommend that peritoneal dissemination may not be a contraindication to the use of bevacizumab. Of note, our data represent exploratory measurements of outcome according to clinical stratifying factors and should be interpreted as such. More data are needed to replicate this interesting result.

Present study represents the first formal evaluation of bevacizumab and cetuximab in the first-line treatment for Chinese mCRC patients. We need to acknowledge that this study is limited by its observational nature. It should also be noted that patients in the bevacizumab group were not specific to *KRAS* wild-type and that the *RAS* status was unknown in this study. Therefore, the degree to which our results can be generalized to *KRAS* or *RAS* wild-type population is unclear.

In conclusion, our study suggests that bevacizumab- and cetuximab-based triplets have similar effectiveness as first-line treatment of mCRC in a Chinese routine practice. Our data additionally raised the possibility that patients with peritoneal dissemination would benefit more from first-line bevacizumab than cetuximab treatment. Thus, our findings indicate a possible new predictive marker that may be helpful in clinical treatment decision-making. Larger prospective studies are needed to confirm these promising results.

## Supplementary Material

Supplemental Digital Content
