# Supplementary material for: Chemotherapy plus bevacizumab versus chemotherapy plus cetuximab as first-line treatment for patients with metastatic colorectal cancer: Results of a registry-based cohort analysis
Source: Medicine (Baltimore). 2016 Dec 23;95(51):e4531. doi: 10.1097/MD.0000000000004531 (PMC5181797; doi:10.1097/MD.0000000000004531)
Supplement: Supplemental Digital Content [file medi-95-e4531-s001.doc]

Table S1. Prognostic value of baseline clinical characteristics s for OS in the bevacizumab group

| Clinical characteristics | Univariate analysis Multivariate analysis | | | |
| --- | --- | --- | --- | --- |
|  | HR (95% CI)*P*HR (95% CI)*P* | | | |
| Sex(female/male) | 1.08 (0.71-1.65) | 0.723 |  |  |
| Age (≤65/>65) | 1.00 (0.99-1.02) | 0.631 |  |  |
| Performance status(0-1/≥2) | 1.54 (1.11-2.14) | 0.017* | 1.56 (1.11-2.21) | 0.011* |
| KRAS status(mutant/wild) | 1.25(0.77-2.02) | 0.364 |  |  |
| Primary tumor(colon/rectum) | 0.85 (0.58-1.25) | 0.417 |  |  |
| Primary tumor(right-sided/left-sided) ^a^ | 0.68 (0.45-1.01) | 0.058 | 1.03 (0.65-1.63) | 0.830 |
| Differentiation(moderately/poorly) | 1.54(1.04-2.28) | 0.031* | 2.01 (1.30-3.11) | 0.002* |
| Metastasis (single/multiple) | 1.25(0.85-1.82) | 0.256 |  |  |
| Backbone regimen (CPT11-based/L-OHP-based) | 1.06(0.74-1.53) | 0.740 |  |  |
| Resection of primary tumor(no/yes) | 0.54 (0.37-0.79) | 0.001* | 0.69 (0.45-1.07) | 0.094 |
| Peritoneal metastasis (no/yes) | 1.11(0.75-1.66) | 0.603 |  |  |
| CEA level^b^ | 2.17 (1.33-3.54) | 0.002* | 2.21 (1.31-3.72) | 0.003* |
| LDH level^b^ | 2.51 (1.68-3.74) | <0.001* | 2.83 (1.81-4.42) | <0.001* |
| ALP level^b^ | 2.45 (1.59-3.80) | <0.001* | 1.20 (0.72-1.99) | 0.483 |
| Disease evolution^c^(synchronous/metachronous) | 0.59 (0.39-0.90) | 0.013* | 0.93 (0.54-1.63) | 0.837 |
| Resection of metastasis sites (no/yes) | 0.31 (0.16-0.59) | <0.001* | 0.25 (0.12-0.54) | <0.001* |

^a^Colon cancers arising in or proximal to the splenic flexure were defined as right-sided colon cancers, and those arising distal to the splenic flexure were defined as left-sided colorectal cancers.^b^Cutoff values: ALP≤ 110 U/L *vs*>110 U/L;CEA ≤ 5 ng/ml *vs*>5ng/ml; LDH ≤ 245 U/L *vs*>245 U/L.^c^ Patients who had metastatic disease after primary tumor resection and less than (synchronous disease) or more than (metachronous disease) six months elapsed fromadjuvant chemotherapy.Abbreviations: OS, overall survival; HR, hazard ratio; 95% CI, 95% confidence interval; CPT11, irinotecan; L-OHP, oxaliplatin;CEA, carcinoembryonic antigen; LDH, lactate dehydrogenase; ALP, alkaline phosphatase. Statistical significance was set at 0.05 based on two-sided test.*P*-values indicated by asterisks were statistically significant.

Table S2. Prognostic value of baseline clinical characteristics s for OS in the cetuximab group

| Clinical characteristics | Univariate analysis Multivariate analysis | | | |
| --- | --- | --- | --- | --- |
|  | HR (95% CI)*P* HR (95% CI)*P* | | | |
| Sex(female/male) | 1.12 (0.71-1.65) | 0.689 |  |  |
| Age (≤65>65) | 1.00 (0.99-1.02) | 0.630 |  |  |
| Performance status(0-1/≥2) | 2.56 (1.46-2.89) | 0.001* | 1.23 (0.61-2.48) | 0.562 |
| Primary tumor(colon/rectum) | 0.56 (0.31-1.00) | 0.050 | 0.73 (0.37-1.43) | 0.767 |
| Primary tumor(right-sided/left-sided) | 0.71 (0.40-1.26) | 0.240 |  |  |
| Differentiation(moderately/poorly) | 1.64(0.88-3.06) | 0.117 | 1.38 (0.74-2.59) | 0.171 |
| Metastasis(single/multiple) | 2.75(1.59-4.73) | <0.001* | 1.38 (0.63-2.99) | 0.683 |
| Backbone regimen (CPT11-based/L-OHP-based) | 1.10 (0.64-1.88) | 0.724 |  |  |
| Resection of primary tumor(no/yes) | 0.35 (0.20-0.64) | 0.001* | 0.58 (0.27-1.23) | 0.788 |
| Peritoneal metastasis (no/yes) | 3.24 (1.66-6.32) | 0.001* | 2.02 (1.08-4.16) | 0.060 |
| CEA level | 1.92 (0.99-3.69) | 0.050 | 1.29 (0.56-2.94) | 0.563 |
| LDH level | 2.68 (1.48-4.85) | 0.001* | 2.12 (1.08-4.16) | 0.029* |
| ALP level | 1.73 (0.92-3.27) | 0.091 | 1.37 (0.61-3.07) | 0.580 |
| Disease evolution^b^ (synchronous/metachronous) | 0.60 (0.34-1.07) | 0.083 | 0.96 (0.46-2.01) | 0.912 |
| Resectionof metastasis sites (no/yes) | 0.31 (0.17-0.59) | <0.001* | 0.26 (0.11-0.62) | 0.002* |

Footnotes as in Supplementary Table 1.

Table S3. Correlations between stratifying factors and clinical outcomesin patients without curative-intent metastasectomy

^a^ Patients were stratified according to baseline stratifying factors, thereafter clinical outcomes of the twosubgroups were compared.^b^ *P*-value for treatment-factor interactionwas assessed by interaction Wald Cox proportional hazards model .In parentheses,the interaction *P*-valuewas adjusted for potential prognostic variables (gender, age, performance status, primary tumor site, tumor grade, prior adjuvant chemotherapy, number of metastasis site, and curative-intent metastasectomy). Abbreviations:Bev, bevacizumab; Cet, cetuximab; Mos, months. Other footnotes as in Table 1.

| Subgroups | PFS (bev group versus cet group) | | | |  | OS (bev group versus cet group) | | | |
| --- | --- | --- | --- | --- | --- | --- | --- | --- | --- |
|  | Median (mos) HR (95%CI) *P* ^a^*P* ^b^ | | | |  | Median (mos) HR (95%CI) *P* ^a^*P* ^b^ | | | |
| All patients | 9.8 v 7.0 | 1.47 (1.09-1.98) | 0.012 | — |  | 24.0 v 20.4 | 1.25 (0.88-1.76) | 0.216 | — |
| Peritoneal metastasis |  |  |  |  |  |  |  |  |  |
| No | 9.8 v 7.7 | 1.30(0.92-1.86) | 0.142 | 0.001 (0.003)* |  | 24.0 v 22.0 | 1.06 (0.71-1.61) | 0.768 | 0.007 (0.030)* |
| Yes | 9.6 v 6.1 | 2.86(1.56-5.24) | 0.001* |  |  | 26.3 v 12.7 | 2.45(1.23-4.85) | 0.010* |  |

Table S4. Incidence of grade 3-4 chemotherapy-related adverse events or serious adverse events

| Adverse events^a^ | All patients | Bevacizumab group | Cetuximab group |
| --- | --- | --- | --- |
| Any grade 3-4 AE^b^ | 119 (41.1) | 77 (41.0) | 42 (41.6) |
| Chemotherapy-related SAE^b^ | 45 (15.6) | 29 (15.4) | 16 (15.8) |
| AE leading to death | 0 (0.0) | 0 (0.0) | 0 (0.0) |
| Neutropenia |  |  |  |
| Grade 3-4 | 71 (24.6) | 45 (23.9) | 26 (25.8) |
| Febrile | 14 (4.8) | 8 (4.3) | 6 (5.9) |
| Nausea | 37 (12.8) | 25 (13.3) | 12 (11.9) |
| Fatigue | 33 (11.4) | 24 (12.8) | 9 (8.9) |
| Vomiting | 35 (12.1) | 25 (13.3) | 10 (9.9) |
| Diarrhea | 32 (11.1) | 18 (9.6) | 14 (13.9) |
| Neurotoxicity | 20 (6.9) | 15 (8.5) | 5 (5.0) |
| Exanthema | 15 (5.2) | 5 (2.7)* | 10 (9.9)* |
| Hand-foot syndrome | 17 (5.9) | 8 (4.3) | 9(8.9) |
| Grade 3-4 transfusion reaction | 3 (0.1) | 1 (0.5) | 2 (2.0) |

^a^The values are presented as median followed by range in parentheses;other values are presented as the number of patients followed by percentages in parentheses. ^b^If a patienthad multiple occurrences of the same event, it was only counted once.Abbreviations: AE,adverse event; SAE, serious adverse event.. *P*-values indicated by asterisks were statistically significant. Valuesindicated by asteriskswere statistically significant differentacrossthe two treatment groups. Abbreviations: AE, adverse event; SAE, Serious adverse event.

Table S5. Incidence of Bevacizumba-Related Adverse Events

| Adverse Events Incidence | |
| --- | --- |
| Bevacizumab-associated SAE | 13 (6.9) |
| AE leading to death | 1 (0.5) |
| Grade 3-4 Hypertension | 12 (6.4) |
| Bleeding event |  |
| Any grade | 22 (11.7) |
| Requiring discontinuation of bevacizumab | 15 (8.0) |
| Grade 3-5 | 7 (3.7) |
| Postoperative wound-healing complication | 1 (0.5) |
| Grade 3-4 Protenuria | 11 (5.9) |
| Venous thromboembolic events | 1 (0.5) |
| Arterial thromboembolic events | 0 (0.0) |
| GI perforation | 2 (1.1) |

Abbreviations:GI perforation, gastrointestinal perforation.Other footnotes as in Supplementary Table S1.
